# Combined Effects of Pericytes in the Tumor Microenvironment

**DOI:** 10.1155/2015/868475

**Published:** 2015-04-27

**Authors:** Aline Lopes Ribeiro, Oswaldo Keith Okamoto

**Affiliations:** Centro de Pesquisa sobre o Genoma Humano e Células-Tronco, Departamento de Genética e Biologia Evolutiva, Instituto de Biociências, Universidade de São Paulo, Rua do Matão 277, Cidade Universitária, 05508-090 São Paulo, SP, Brazil

## Abstract

Pericytes are multipotent perivascular cells whose involvement in vasculature development is well established. Evidences in the literature also suggest that pericytes display immune properties and that these cells may serve as an *in vivo* reservoir of stem cells, contributing to the regeneration of diverse tissues. Pericytes are also capable of tumor homing and are important cellular components of the tumor microenvironment (TME). In this review, we highlight the contribution of pericytes to some classical hallmarks of cancer, namely, tumor angiogenesis, growth, metastasis, and evasion of immune destruction, and discuss how collectively these hallmarks could be tackled by therapies targeting pericytes, providing a rationale for cancer drugs aiming at the TME.

## 1. Introduction

It has become increasingly evident that, not only the evolving genetic aberrations in malignant cells are critical in the pathophysiology of cancer, but also the interaction among cancer cells, nonmalignant cells, soluble factors, and other elements of the tumor microenvironment (TME). In addition to cancer associated-fibroblasts, immune cells, and endothelial cells (ECs), pericytes are also one of the main cellular components of the TME, whose diverse functions in tumor initiation and progression have only been recently addressed [1].

Pericytes were first described in the 19th century, at that time named “adventitial cells” by Rouget [2]. The term “pericyte” would only be applied in 1923, by Zimmermann [3]. These cells are commonly located on microvessel walls, within the basement membrane and closely opposed to the endothelium.

Under the microscope, pericytes are typically described as highly elongated, slender, and branched cells, with projections that extend longitudinally and circumferentially around the vessel wall [4, 5]. Pericytes have also been characterized by the expression of alpha-smooth muscle actin (*α*-SMA), desmin, CD146, platelet-derived growth factor beta receptor (PDGFR*β*), and nerve/glial antigen-2 (NG2) proteoglycan [5, 6]. These markers, however, are not exclusive of pericytes and their expression may also vary according to the type of tissue, maturation stage, and pathological conditions [5, 7]. The use of different markers or combination of markers varies in the literature and, so far, a consensus about the phenotypic identity of pericytes has not been reached. Nonetheless this issue needs to be considered to better understand pericyte biology.

For instance, in a study using double transgenic Nestin-GFP/NG2-DsRed mice, Birbrair et al. [8] identified two pericyte subpopulations from large blood vessels and small capillaries, named type-1 and type-2 pericytes. These cell subpopulations expressed common pericyte markers, such as PDGFR*β*, CD146, and NG2, but differed in Nestin expression. These distinct pericyte subtypes were later functionally characterized and shown to differ in their multipotent properties [8, 9] and angiogenic potential [10].* In vitro* and* in vivo* assays revealed that type-2 (Nestin-GFP+/NG2-DsRed+), but not type-1 (Nestin-GFP−/NG2-DsRed+), pericytes are recruited during tumor angiogenesis. However, little is known about the ontogeny of these distinct subtypes and whether they are interconvertible.

In fact, the essential contribution of pericytes to vasculature development and maintenance has long been known. They participate in the regulation of blood flow and vessel permeability, as well as in stabilization of the vascular wall [11]. Pericytes also provide important mechanical and physiological support to ECs and such interaction is essential for vessel remodeling and maturation [12, 13]. More recently, there have been growing evidences supporting new roles for pericytes in immunomodulation [14] and adult stem cell biology [15].

In the context of cancer, these distinctive pericyte properties make them important modifiers of disease progression, contributing directly or indirectly to tumor growth, metastatic spread, and resistance to therapy.

## 2. Tumor Angiogenesis

Tumor-driven angiogenesis was first described more than 100 years ago [16]. The later observation that without an efficient blood supply tumors could not grow beyond a critical size or metastasize stimulated an intensive search for pro- and antiangiogenic molecules [17, 18]. Nowadays, some of the latest therapeutic options for treatment of different cancers rely on antiangiogenic strategies, such as bevacizumab, a monoclonal antibody targeting vascular endothelial growth factor (VEGF).

Angiogenesis is a multiple-step process tightly orchestrated by many molecules that regulate both ECs and pericytes activities. Pericytes are known to secrete growth factors that stimulate EC proliferation, in addition to proteases that contribute to modulate the surrounding extracellular matrix and guide EC migration [13, 19–21]. The proliferative endothelium from a preexisting structure with the basement membrane forms an initial tube still in an immature state. Subsequently, ECs release signals that induce pericyte recruitment [22]. The resulting pericyte coverage is crucial for vessel remodeling, maturation, and stabilization.

The reciprocal communication between ECs and pericytes is established by direct contact, by paracrine signaling, or by a newly described chemomechanical signaling pathway [23]. Some of the signaling molecules involved in this crosstalk coordination include angiopoietin-1/2 and Tie2 (Ang/Tie2), transforming growth factor-*β* (TGF-*β*), and platelet-derived growth factor-*β* (PDGF*β*/PDGFR-*β*), which are mainly related to EC viability, mural cell differentiation, and pericyte recruitment, respectively [24].

Similar events occur during tumor angiogenesis. The sprouting of ECs is followed by a pericyte migration but, in this case, the vascular architecture does not accomplish complete maturation, which leads to several structural and functional abnormalities [25, 26]. Tumor vessels are highly disorganized, irregularly shaped, tortuous, excessively branched, and leaky [27]. The basement membrane is discontinuous or absent and presents altered composition [28]. The endothelium can be incomplete or occasionally multilayered. ECs and perivascular cells also differ functionally and morphologically from their normal counterparts [29, 30]. In tumor vessels, pericytes are loosely attached to the endothelium and exhibit cellular modifications such as differential expression of typical markers and aberrant cytoplasmic projections that invade the tumor parenchyma [31–33].

**Figure 1:**
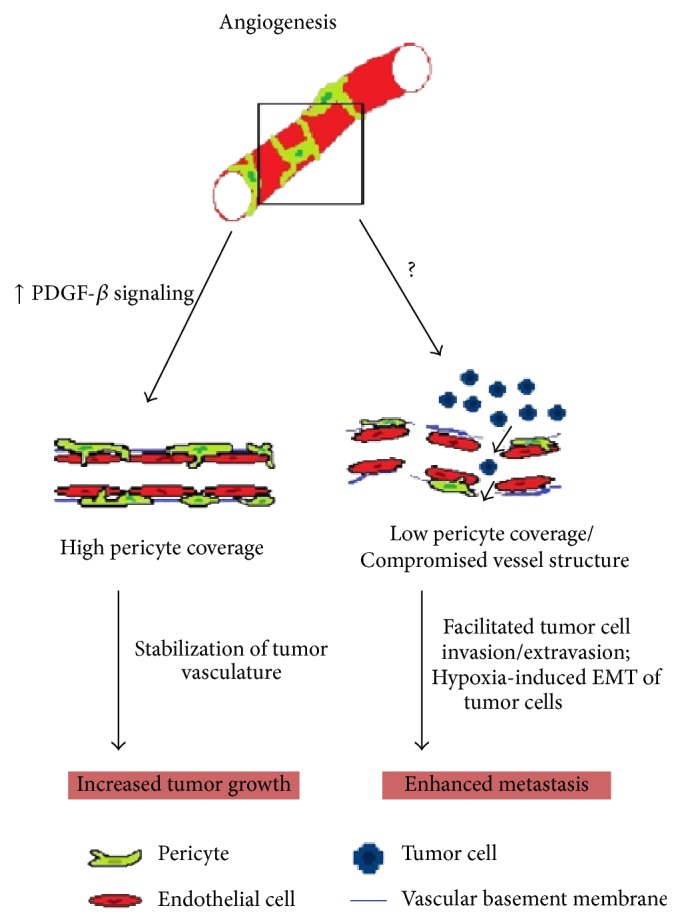
Abnormal pericyte coverage of tumor vessels affects tumor development. PDGF-*β* signaling controls pericyte recruitment during angiogenesis. Hyperactivation of this pathway within TME may increase pericyte coverage, thereby improving vasculature stability and perfusion, which favors tumor growth. In contrast, low pericyte coverage compromises vessel structure integrity, which becomes leaky, facilitating tumor cell invasion/extravasion. Under such circumstances, tumor cells may also undergo EMT induced by hypoxia, as a consequence of lower perfusion in the tumor vasculature. Both situations enhance metastatic spread of tumor cells.

Another abnormality often observed in tumor angiogenesis is the amount of pericyte coverage on tumor vessels, ranging from high to little or no coverage at all. Clinical studies have correlated the extension of pericyte coverage on tumor microvessels with cancer prognosis [34–37]. Increased pericyte coverage has been associated with tumors of melanoma and renal cell carcinoma with aggressive clinicopathological features, resistance to therapy, and unfavorable clinical outcome of patients [38]. In contrast, pericyte dysfunction or reduction has not been correlated with prognosis. Recent studies reported that pericyte ablation leads to increased vessel permeability and poor vessel integrity which, in spite of inhibiting tumor growth, favors blood vessel invasion by tumor cells and ensuing metastatic spread [39, 40]. These findings illustrate the many facets of pericyte effects on tumor angiogenesis (Figure 1).

It is still unclear why tumor vessels are not able to achieve proper pericyte coverage. One important mechanism of EC-pericyte communication involves the PDGF-*β* signaling, which is known to control pericyte migration during tumor angiogenesis [41]. In such mechanism, activated ECs produce PDGF-*β*, recruiting pericytes expressing PDGF-*β* receptors [22, 42]. In turn, pericytes stabilize the neovessels and contribute to ECs survival by locally releasing trophic factors, such as VEGF and Ang-1 [43, 44]. Blockage of pericyte recruitment by PDGF-*β* pathway inhibition leads to EC loss and subsequent regression of tumor vessels [30, 45]. Overexpression of PDGF-*β*, on the other hand, increases pericyte coverage, improves vessel stability, and accelerates tumor growth rates [46, 47]. Due to its relevance, therapies targeting pericyte recruitment have been considered. Other mechanisms governing pericyte migration have been covered in a recent review [48].

Pericytes have been shown to be capable of providing a scaffold of preexisting blood vessels for rapid revascularization of tumors after interruption of therapies that eliminates only ECs [49]. It seems that the remaining pericytes participate in a strategy developed by tumors to evade antiangiogenic therapies. Consequently, the combination of anti-VEGF and anti-PDGF therapies has been proposed and was shown to induce tumor vessel regression [50, 51]. More recently, treatment with anti-OLFML3 (olfactomedin-like 3) was reported to be significantly effective in reducing tumor vascularization, pericyte coverage on tumor vessels, and tumor growth [52].

Therefore, in addition to their role in tumor angiogenesis, the involvement of pericytes in vessel cooption, an important alternative pathway by which tumors obtain blood supply through the use of preexistent vessels, supports the development of novel antiangiogenic strategies targeting, not only ECs as usual, but also pericytes. The proposal that interaction of pericytes with tumor cells may determine the perivascular location of tumor propagating cells [53] provides further arguments to the relevance of pericytes in tumor development, although details of this phenomenon remain to be determined.

## 3. Metastasis

Dissemination of cancer cells to distant organs requires their survival through a challenging route beginning in the primary tumor site. Invasion into surrounding vessels or tissues, survival in a hostile environment (e.g., blood circulation), and ability to seed and recapitulate tumor growth in a new site are the main limiting steps in this process. All stages can be highly influenced by nonmalignant cells within the tumor microenvironment, including pericytes.

Although the initial studies of pericytes and tumor development were mostly focused on angiogenesis, showing that blockage of pericyte recruitment or function leads to reduced tumor growth due to compromised vessel structure and blood supply [26], later studies surprisingly revealed that loss of pericyte coverage facilitates tumor cell spreading.

One of the first evidences showing that pericytes may be negative regulators of metastasis was provide by Xian et al. (2006), using mice deficient in neural cell adhesion molecule (NCAM). In this landmark paper, they provide compelling evidence that destabilization of tumor vasculature due to detachment of pericytes and dysfunctional interaction with ECs leads to enhanced metastatic potential [54]. A previous study had already observed an enhanced metastatic frequency in knockout animals exhibiting compromised blood vessel structure [55]. Further clinical studies with colorectal and breast cancer patients corroborated this finding [35, 40]. Low pericyte coverage showed a significant correlation with distant metastasis and poorer survival. Similarly, in a xenograft model of prostate cancer, increased tumor cell invasion was associated with lower pericyte density on microvessels [56].

However, the underlying cellular and molecular mechanisms whereby pericytes may limit tumor metastasis have not been entirely elucidated. Pericytes may act as a physical barrier that makes the extravasation of tumor cells into the vessel lumen difficult and/or may actively promote metastasis by releasing factors that affect tumor invasion.

Alternatively, a recent proposal defends the idea that pericytes may be indirectly involved in tumor cell escape. They hypothesize that pericyte depletion originates leaky vessels which increases intratumoral/interstitial plasma volume and elevates local pressure. The higher fluid pressure favors compression of remaining tumor vessels, decreasing the blood flow and reinforcing hypoxia, which may trigger tumor metastasis through a hypoxia-induced epithelial-mesenchymal transition (EMT) mechanism [40]. In fact, recovery of tumor vascular integrity by improving ECs junctions and increasing pericyte coverage was effective in reducing leakage and enhancing perfusion. In melanoma models, normalization of tumor vessels was able to attenuate hypoxia and decrease EMT of tumor cells, resulting in inhibition of lung and lymph node metastasis [57].

Pericytes have also been suggested to contribute to the metastatic process by affecting the colonization and growth of tumor cells at distant sites [9]. The contact of tumor cells with microvessel walls in a pericyte-like position seems to be determinant in the successful extravasation and proliferation of melanoma and lung carcinoma cells in the brain [58]. Studies with two murine models of lung metastasis showed that the administration of sunitinib, a clinically approved antiangiogenic drug, led to pericyte depletion in the seeding location [59]. Interestingly, tumor cells were preferentially retained in the lung vasculature area displaying lower pericyte coverage. The suggested hypothesis is that pericytes may limit seeding at the target site, controlling and regulating the metastatic niche.

Moreover, endothelial-derived factors have been reported to influence breast cancer cell growth at sprouting vessels in metastatic sites [60]. Taken together, these findings support the emerging idea that microvascular cells, including pericytes, may affect metastasis establishment and tumor cell growth at secondary sites.

## 4. Stemness

The multipotent differentiation capacity of pericytes has long been proposed. In 1978, Meyrick and Reid had already demonstrated that pericytes were plastic cells, capable of developing into vascular smooth muscle cells (vSMCs) under hypoxic stress [61]. Differentiation into other nonvascular cells, primarily bone cells, was later described [62]. Subsequently, several studies described that pericytes obtained from a variety of tissues could differentiate into adipocytes, chondrocytes, and skeletal myofibers [63, 64]. Furthermore, pericytes derived from brain capillaries were also reported to be capable of converting into neural cell lineages [65]. These and several other evidences support the hypothesis of the perivascular zone as the* in vivo* niche of mesenchymal stem cells (MSCs) and pericytes being the MSC precursors [66].

Indeed, besides multipotency, pericytes and MSCs share other similarities, including expression of common cellular markers. While pericytes express surface antigens typical of MSCs, such as CD44, CD73, CD90, and CD105, MSCs also express pericytes markers, including NG2, Sca-1, *α*-SMA, and PDGF*β*-R [15], suggesting a shared ontogeny. Both cell types also present similar homing properties. Pericytes and MSCs can proliferate and migrate in response to chemotaxis and damage signals, such as those occurring during wound healing and tumor development.

Some recent findings, however, indicate that not all pericytes display stem cell potential, such as the highly differentiated pericytes found in some large and small vessels [67, 68]. Other studies also suggest that pericytes may be a subpopulation of specialized MSC residing in perivascular locations, given that the pericytic behavior is not an intrinsic ability of all MSCs [68].

There are also growing evidences of pericytes with stemness potential in several central nervous system pathologies. Compared with other tissues, the pericyte number and coverage in brain capillaries are relatively higher, and they are crucial to the integrity and function of the blood-brain barrier. Pericytes have been considered as an alternative stem/progenitor cell reservoir within the brain, since they were shown to migrate, proliferate, and even differentiate in neural cells, in response to tissue injury, stress, and inflammation [65, 69, 70].

In brain cancer, the perivascular niche is critical to the maintenance of a stem cell-like state in tumor cells. Interaction of perivascular cells with cancer stem cells (CSC) was shown to regulate self-renewal and differentiation of the latter cells, which are strongly related to tumor aggressiveness [71]. Notably, in Glioblastoma, the most frequent and aggressive type of primary brain tumor, a contact-dependent interaction with tumor cells, switches on the tumor-promoter character of pericytes, inducing their participation in tumor initiation and progression [53].

**Figure 2:**
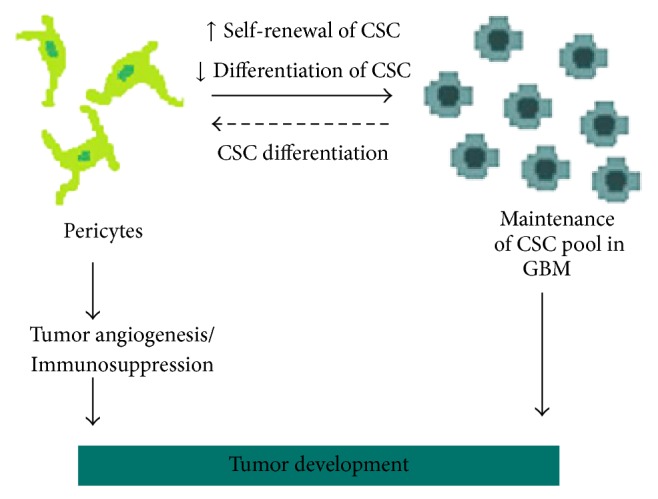
Interplay between pericytes and cancer stem cells. In brain cancer, the perivascular niche is critical to the maintenance of CSC pool. Perivascular cells promote self-renewal and impair differentiation of CSC. In turn, new pericytes may be generated by CSC, contributing to tumor angiogenesis and tumor scape from immune destruction. This reciprocal interaction between pericytes and CSC is highly beneficial to tumor development.

Another surprising connection between pericytes and brain CSC was revealed by Cheng et al. (2013) [72]. The authors demonstrated that most pericytes residing in the perivascular niche of Glioblastoma are generated by CSC. Through a close interaction with vascular components, Glioblastoma stem cells are also able to diferentiate into functional endothelial cells [73]. These findings reveal an interesting reciprocal interaction between pericytes and CSC, favoring tumor development (Figure 2).

Based on the MSC properties of pericytes, Appaix et al. [74] also proposed that neoplastic pericytes in brain capillaries could be activated and recruited in response to inflammation signals, similar to what occurs during tissue regeneration. These neoplastic pericytes would then acquire a neural stem cell-like phenotype in the brain parenchyma and generate a pool of CSC, fueling tumor development. New pericytes generated from CSC could either contribute to tumor vascularization or restart the cycle. Due to their multipotency, pericytes could also generate other stromal cells constituting the TME. In fact, pericytes have been shown to differentiate into collagen-producing fibroblasts [75] and myofibroblasts [76], two major components of the heterogeneous population of cancer-associated fibroblasts. Although plausible, further experimental evidences are needed to support this model of neoplastic pericytes as tumor initiating cells.

In addition to inflammation signals, hypoxia is another important extrinsic factor within the TME that may recruit pericytes. Interestingly, brain-derived pericytes have been recently reported to generate neurovascular cells and activated microglial cells under hypoxic conditions [77, 78]. In gliomas, the most frequent group of primary central nervous system tumors, microglial cells are known to be recruited to the TME and activated to support tumor growth [79, 80]. Altogether, these evidences support an important role of pericytes as precursor cells for other stromal cells within the TME.

## 5. Immunomodulation

Tumor cells can evade the immune system through different mechanisms, some of which involving multiple cellular components and immunosuppressive factors (e.g., TGF-*β*, prostaglandin E2, and interleukin-10) from the TME. Although the contribution of pericytes in this process is still elusive, recent data support pericytes as potential targets in cancer immunotherapy approaches.

Similar to MSCs, pericytes produce cytokines, chemokines, growth factors, and adhesion molecules that regulate immune cells under certain conditions. Several genes encoding immune factors in pericytes have been reported to be upregulated by activation of the PDGF-*β* signaling pathway [81], whose involvement in pericyte migration during angiogenesis was described above. In fact, pericytes have been considered an important component of the immunologic defense mechanism in the mammalian central nervous system [82, 83], where they were reported to express typical macrophage markers, such as ED-2, CD11b, CD68, and MHC class II, and exhibit immune cell properties, such as phagocytic and antigen-presentation activities [14, 84].

Brain pericytes, in particular, are highly sensitive to inflammatory stimuli and may differentially respond according to the cytokine involved. Studies with porcine brain capillary pericytes reported a rapid upregulation of iNOS and COX-2 mainly after stimulation with interleukin-1 beta (IL-1*β*). Upregulation of iNOS was accompanied by increments in the intracellular oxidative status of pericytes. The same study also reported induction of phagocytosis of opsonized particles and MHC II expression in pericytes by tumor necrosis factor-alpha (TNF-*α*) or interferon-gamma (IFN-*γ*) treatment, characteristic of an antigen-presentation activity [84].

Interaction between pericytes and immune cells also occurs during tissue repair, when pericytes may actively participate in leukocyte recruitment and diapedesis. Using an experimental model of brain inflammation, Pieper et al. [85] demonstrated that treatment with TNF-*α*, IL-1*β*, or LPS stimulates secretion of IL-8 and matrix metalloprotease-9 by brain pericytes, facilitating chemoattraction and transmigration of neutrophils.

However, in the cancer context, there are evidences that maturation of pericytes and restoration of the normal tumor vasculature improve transmigration of immune cells into tumors. A study with the RIP1-Tag5 mouse model of pancreatic islet carcinogenesis showed that deletion of the* Rgs5* gene, encoding a regulator of G-protein signaling with expression restricted to pericytes in the vascular tissue, induced changes in the vasculature and enhanced infiltration of CD8^+^ T lymphocytes in tumors. As a consequence, the immune-mediated tumor rejection was exacerbated, resulting in improved survival of tumor-bearing mice [86].

In agreement with these observations, Bose et al. [87] reported upregulation of* Rgs5* in murine pericytes when these cells were cocultured with fragmented tumor cells or were directly injected into established tumors* in vivo*. Moreover, tumor-derived pericytes were able to induce CD4+ T cell anergy and this effect was rescued after* Rgs5* silencing. Interestingly, in addition to* Rgs5*, upregulation of* PDL-1* was also observed in pericytes cultivated in the presence of tumor fragments. Since PDL-1 expression in cancer cells is known to inhibit the activity of PD-1+/CD8+ T cells [88], the combined effects of RGS5 and PDL-1 expression in pericytes may improve protection of tumor cells from T cell-mediated death. Indeed, pericytes isolated from human malignant gliomas, characterized by coexpression of CD90, PDGFR-*β*, and CD248, were also suggested to have immunosuppressive properties within the TME, based on their capacity to inhibit proliferation of cytotoxic T lymphocytes [89].

These findings point out important direct and indirect effects of pericytes in the immune response against tumor cells, whose underlying mechanisms remain to be fully dissected.

## 6. Conclusions

An overall analysis of the functional properties of pericytes reveals that these are multifaceted cells with ability to significantly influence tumor development. As a component of the TME, pericytes may actively contribute to some classic cancer hallmarks, namely, induction of angiogenesis, sustained tumor growth, metastasis, and evasion of immune destruction.

Disruption of the delicate balance of pericyte coverage on tumor vessels seems critical since it may either induce tumor growth or facilitate metastatic spread. The interplay between pericytes and CSC is also compatible with the updated dynamic CSC model. Pericyte-mediated regulation of stemness properties in cancer cells could help maintain a residual CSC pool, whose cell progenies include both tumor and stromal cells. The immunosuppressive phenotype acquired by pericytes once in the TME is also of great relevance since they may act in synergy with tumor cells to inhibit local immune response. This scenario is highly favorable to current cancer immunotherapy strategies, such as the use of monoclonal antibodies targeting the PD-1/PDL-1 signaling.

Altogether, this analysis argues in favor of pericytes as cellular targets for new cancer therapies aiming at the TME. Modern cancer treatments largely rely on such strategy, with antiangiogenic and immunosuppressive drugs as the main examples. Development of new drugs addressing pericytes would have the advantage of targeting multiple cancer hallmarks at once, increasing the chances of treatment efficacy. However, given the prometastatic effects of pericyte depletion on tumor vessels, development of such therapeutic strategy is not straightforward and should be more beneficial to early-stage diseases or to tumors with low metastatic potential. As phenotypic and functional characterization of pericytes progresses, particular subtypes of pericytes may also emerge as clearer targets for therapeutic purposes, such as the case of type-2 pericytes which are specifically recruited during tumor angiogenesis. Ultimately, pericyte-targeted therapies should be tested in combination with other treatment modalities to address possible synergistic effects aiming at meaningful tumor regression without favoring metastatic spread.
